# Supplementary material for: A Global Screen for Assembly State Changes of the Mitotic Proteome by SEC-SWATH-MS
Source: Cell Syst. 2020 Feb 26;10(2):133–155.e6. doi: 10.1016/j.cels.2020.01.001 (PMC7042714; doi:10.1016/j.cels.2020.01.001)

Q9BT22 | ALG1_HUMAN | ALG1 HMAT1 HMT1 PSEC0061 UNQ861/PRO1870
Monomer MW [kDa]: 52.518 Monomer expected elution fraction: 46

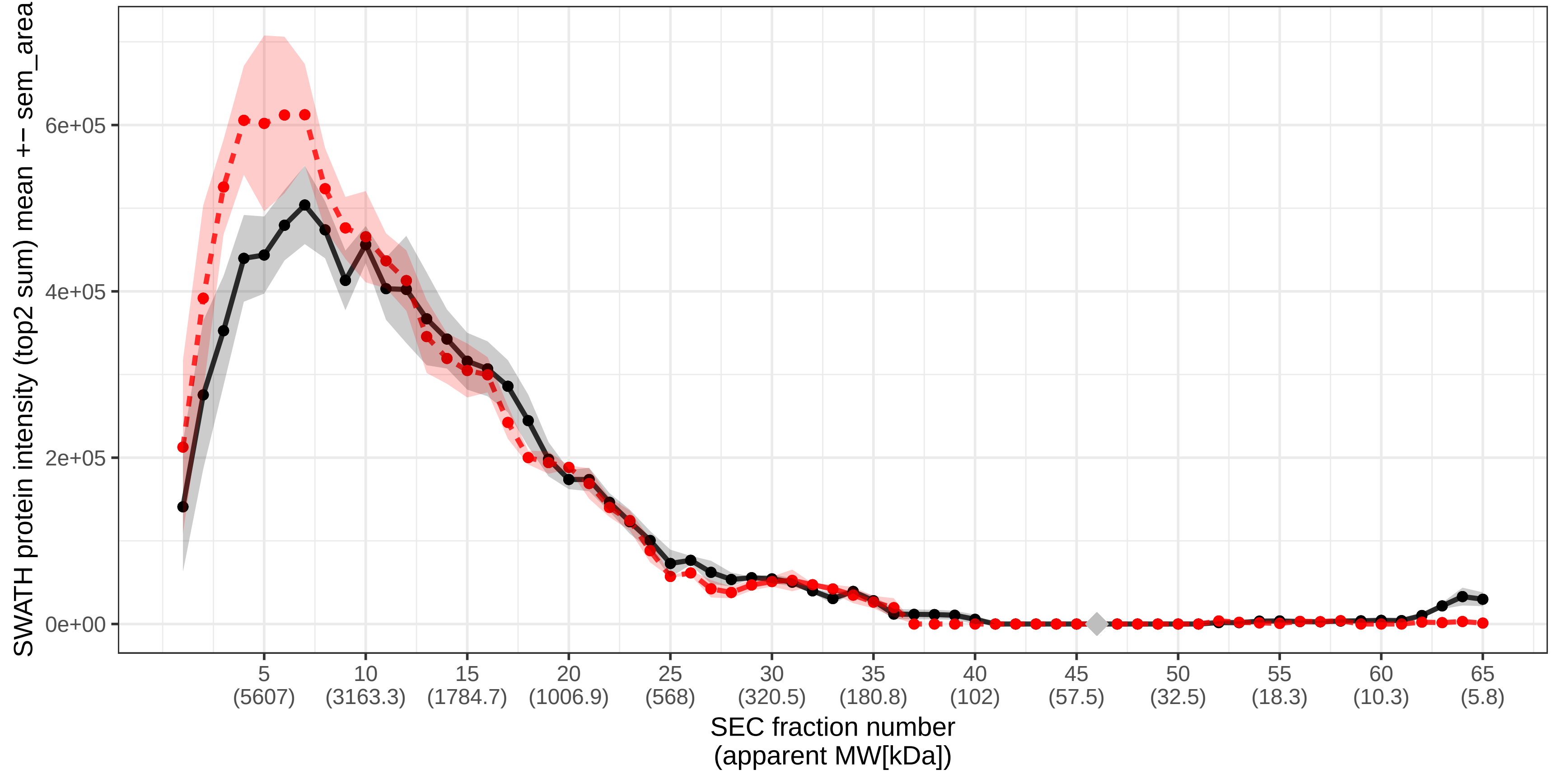

Supplement: Data S1. SEC-SWATH-MS Protein Chromatograms, Related to Figure 1 [file mmc6.zip › SECchrom_Q9BT22_ALG1_HUMAN_ALG1_HMAT1_HMT1_PSEC0061_UNQ861_.pdf]
